# A Trauma-Informed, Family-Centered, Virtual Home Visiting Program for Young Children: One-Year Outcomes

**DOI:** 10.1007/s10578-021-01181-y

**Published:** 2021-05-07

**Authors:** Catherine Mogil, Nastassia Hajal, Hilary Aralis, Blair Paley, Norweeta G. Milburn, Wendy Barrera, Cara Kiff, William Beardslee, Patricia Lester

**Affiliations:** 1 Jane and Terry Semel Institute for Neuroscience and Human Behavior, University of California Los Angeles, 760 Westwood Plaza, Room A8-225, Los Angeles, CA 90024 USA; 2 Private Practice, Los Angeles, CA USA; 3 Baer Prevention Initiatives, Boston Children’s Hospital, Boston, MA USA

**Keywords:** Military-connected families, Telehealth, Family-centered prevention, Family resilience, Parenting

## Abstract

Military-connected families face many challenges associated with military life transitions, including deployment separations. We report on a randomized controlled trial to evaluate the efficacy of Families OverComing Under Stress-Early Childhood (FOCUS-EC) delivered through an in-home, virtual telehealth platform. FOCUS-EC is a trauma-informed, family-centered preventive intervention designed to promote family resilience and well-being. Military-connected families with 3- to 6-year-old children (194 mothers; 155 fathers; 199 children) were randomized to FOCUS-EC or an online education condition. Parent psychological health symptoms, child behavior, parenting, and parent–child relationships were examined by parent-report and observed interaction tasks for up to 12 months. Longitudinal regression models indicated that FOCUS-EC families demonstrated significantly greater improvements than online education families in parent-reported and observational measures of child behavior, parenting practices, and parent–child interaction, as well as greater reductions in parent posttraumatic stress symptoms. Findings provide support for the benefit of a virtually-delivered preventive intervention for military-connected families.

## Introduction

Parental military service affects not only service member well-being, but also that of their children [1–4]. About half of children in military-connected families (MCF) are aged 0–6 years in the U.S. [1, 2] and Canada [3]. Early childhood represents a critical period when development is unfolding at a rapid pace and parents are necessarily helping their children achieve critical physical, cognitive, social and emotional milestones [4]. While there are multiple pathways by which parent military service affects development in young children, research highlights the importance of parental psychological well-being and parenting behaviors in predicting young children’s adjustment [5–7]. This study reports on the results of a randomized controlled trial of the Families OverComing Under Stress for Early Childhood program (FOCUS-EC; [8]), a trauma-informed, family-centered preventive intervention delivered to military-connected families with young children through a virtual telehealth platform.

Research indicates that most children adapt well to life in a military family [5, 9]. In fact, there are certain aspects of military life that are conducive to family resilience [10]. Yet, there are challenges to communication, family cohesion, routines and roles that can accompany a parent’s military service (for review, see [7]). For example, during military-related family separations (e.g., due to deployment, non-local military-related training, etc.), the at-home parent may experience a range of difficult emotions, including fear, anxiety, loneliness, and frustration related to a variety of stressors such as increased responsibilities in the home, temporary loss of a co-parent, and worry about their partner’s safety [11]. Increased psychological distress may render the at-home parent less able to support their child in the midst of the child’s own worry about their deployed parent and stress associated with separation from an attachment figure [12]. Research on military-related family separations has focused primarily on deployment separations. Prior studies have found that parental deployment may be especially challenging for families with young children, as suggested by the finding that young children are at higher risk than older children of maltreatment over the course of deployment [13].

Parental military service experiences may present risk for families, even after a military-related separation ends. Posttraumatic stress, depression, anxiety, and substance abuse symptoms are documented to be as high as 30% in some military and veteran samples [14, 15], and can have cascading negative effects on parenting and family functioning. There is robust literature, both civilian and military, linking parent mental health symptoms with maladaptive parenting behaviors [16], which then contribute to child maladjustment [17, 18]. Given that young children’s development is so deeply embedded within parent–child relationships, children aged 0–6 years can be especially vulnerable to their military parents’ mental health problems [19]. Furthermore, the dependence of young children on their parents for external regulation of emotion and behavior may be especially taxing for parents who are already experiencing baseline levels of emotional dysregulation and reactivity themselves. Two independent samples of families with young children showed that previously deployed parents’ posttraumatic stress disorder (PTSD) symptoms were associated with children’s emotional and behavioral problems and parents’ perceptions of dysfunctional parent–child interactions [20, 21]. These associations are not unique to service member parents; non-deployed parents’ depressive and PTSD symptoms after a deployment were also associated with young children’s socioemotional development and anxiety symptoms [22, 23].

These findings indicate that preventive interventions designed to enhance children’s adjustment in the face of parental military service should focus on the psychological health of all caregivers and the functioning of the family system, including parenting and parent–child relationships. Despite established research demonstrating the benefits of family-centered preventive interventions for at-risk families [16, 17], military-informed family-centered services for MCF, particularly those living in civilian communities, are limited due to a range of barriers. Research indicates that even for active duty families eligible to receive child and family services through the Department of Defense (DOD), almost 40% of active duty parents seek services for their young children from non-military providers [24]. National Guard and Reserve families have limited access to DOD services; these parents reported seeking services from non-military pediatricians and schools when concerned about their young children’s adjustment [24]. The Veteran Health Administration (VHA) does not provide services for children, although efforts to provide couples and family counseling and parenting support have recently been initiated at some individual VHA locations [25]. Thus, most community-dwelling MCF must access child and family services within civilian settings, which may lack specific training in military family culture and well-being (although there have been increasing efforts to enhance the capacity of community providers to serve MCF through professional and community-based training, e.g., [26, 27]). Just as for civilian families with young children, MCF often have limited time and resources, further limiting access to prevention services. Thus, there is clearly a need for more accessible, family-centered interventions that are sensitive to military culture.

Studies of two interventions, ADAPT (After Deployment, Adaptive Parenting Tools; [28]) and Strong Families, Strong Forces (SFSF; [29]), have shown support for improving parenting practices in MCF families. Each intervention focuses on a unique period of deployment (i.e., after deployment) or has been used with a subsector of military families (i.e., National Guard). Both aim to reduce barriers for families to access care [7]. SFSF uses a home visiting model, which is appropriate for local National Guard/Reserve families; however, the costliness of in-person home visiting services poses a challenge for many community providers and clinical systems. A third intervention, Families OverComing Under Stress (FOCUS), has been developed to promote family-level resilience and psychological well-being in military and veteran families facing the many transitions associated with military life, including separations due to trainings, parental deployments and transition to veteran status. FOCUS has been implemented at scale at more than 30 U.S. military installations using a tiered public health approach [30, 31] and has been adapted for office-based and in-home delivery formats. Previous program evaluation with active duty families with school aged and adolescent children has shown that FOCUS participants demonstrate improved parent, child, and family adjustment and that these improvements are maintained at 6 months post-intervention [32]. In response to an identified need, FOCUS was adapted to be suitable for use with MCF families with a preschool-aged child [8], utilizing the Center for Disease Control’s adaptation framework [33]. Using this model, we maintained fidelity to our core elements while customizing key characteristics to be responsive to needs of families with young children (see [7, 34, 35] for detailed descriptions of the adaptation framework). Due to the geographic dispersion of MCF families [25] and usefulness of home-visiting models for families with young children (e.g., [36, 37]), FOCUS-EC was adapted to be delivered as a virtual home visiting model. A feasibility pilot comparing the in-person home-visiting and virtual home-visiting model indicated that a fully virtual home-visiting approach was feasible and acceptable for MCF [8].

The present study builds upon previous program evaluation support for FOCUS in three important ways: (1) evaluating the adapted intervention for MCF with young children; (2) assessing a virtual home-visiting approach to delivering services to community-dwelling MCF; and (3) testing the intervention in a randomized controlled trial, with three follow-up assessments over the course of a year. Outcomes assessed included parents’ reports of their own psychological health symptoms, parenting stress and behavior, and parent–child relationships. Furthermore, standardized parent–child interaction tasks were video-recorded and coded to capture a more objective measure of parent and child behaviors and parent–child relationships. We hypothesized that families randomized to receive the FOCUS-EC intervention would demonstrate significantly greater improvements from baseline to 3-, 6-, and 12-month follow-ups on measures of parent psychological symptoms, parenting and parent–child relationships, and child behavior, relative to families randomized to a standardized online parenting education (OPE) curriculum, which served as the control condition.

## Method

### Intervention

FOCUS-EC is a trauma-informed, family-centered preventive intervention for families with young children and is designed to be flexibly customized to fit each family’s unique goals and challenges. Due to geographic dispersion of MCF families [25] and usefulness of home-visiting models for families with young children [36, 37], FOCUS-EC was adapted to be delivered via virtual home-visiting using a telehealth platform as described previously [8].

**Fig. 1:**
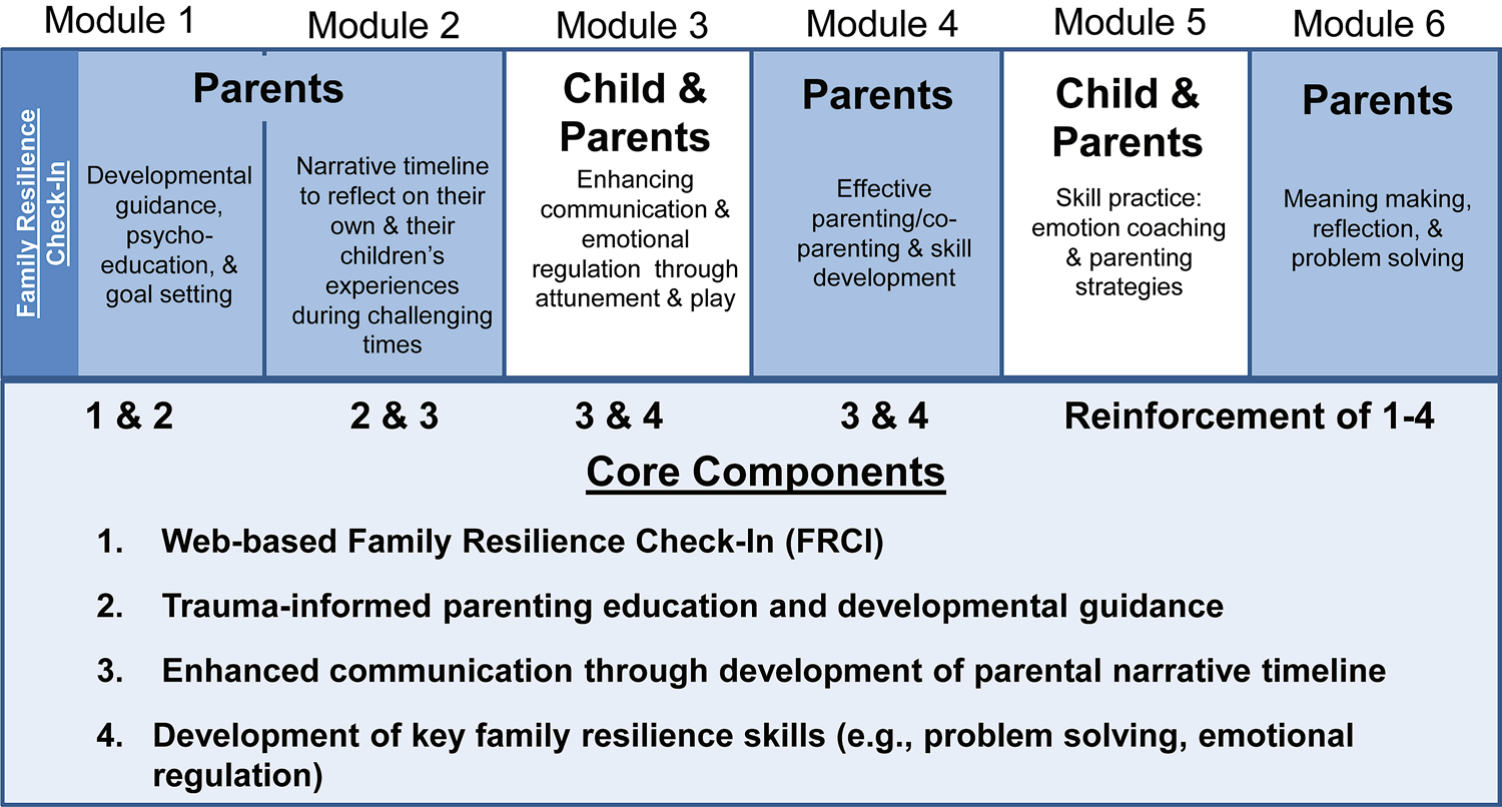
FOCUS-EC session overview

FOCUS-EC consists of core elements delivered in 6 modules that are typically delivered over 4–10 meetings that last 60–90 min. Consistent with the FOCUS model described previously [34, 35], the core elements include (1) web-based Family Resilience Check-In (FRCI); (2) personalized trauma-informed psychoeducation, parenting education and developmental guidance; (3) development of a parental narrative timeline to support reflection, empathy, meaning making and communication; and (4) development of family resilience and parenting/co-parenting skills. At the beginning of FOCUS-EC, parents complete the FRCI, which consists of standardized assessments and real-time feedback of parent psychological health symptoms, child socioemotional functioning, and family adjustment. The FRCI is used to tailor developmental guidance and psychoeducation throughout the remainder of FOCUS-EC, as well as to personalize skill development. A description of FOCUS-EC has been published elsewhere [8] and can be seen in Fig. 1.

FOCUS-EC was delivered by doctoral or master’s level mental health providers with experience delivering child and family interventions. Supervisors reviewed cases weekly during individual and group supervision, read progress notes, and periodically co-facilitated or observed sessions, which were video-recorded. Approximately 20% of recorded sessions were coded for intervention fidelity by trained coders, who rated interventionists’ adherence to the core actions of the FOCUS-EC model on a scale of 1 (Yes/Completed) to 3 (No/Not completed). The overall mean of interventionist fidelity was excellent (M = 1.31, SD = 0.22). When clinical risk was identified (e.g., suicidal ideation), further screening, emergency management and appropriate treatment referrals were implemented.

The active control condition included access to online parent education (OPE). The OPE condition consisted of educational resources for military-connected parents of young children presented in four asynchronous self-guided modules: (a) Positive Parenting, (b) Supporting Child Development, (c) Maintaining Self-Care, and (d) Navigating Family Transition. These modules included high-quality resources for military-connected parents of young children curated from sources such as the U.S Department of Veterans Affairs’ Parenting for Veterans, Sesame Street for Military Families and American Academy of Pediatrics’ Healthy Children. While the modules were consistent with parenting education and developmental guidance provided in the FOCUS-EC program’s educational core content, they did not include the FOCUS core intervention components.

The University of California Los Angeles Institutional Review Board approved this study.

### Recruitment

Families with a child aged 3–6 years with at least one parent who served post-9/11 in the US Army, Navy, Marine Corps or Air Force were recruited through targeted social media advertising, at military- and veteran-serving events and organizations and through word-of-mouth community referrals in Southern California. In order to participate, the service member/veteran parent had to be available for assessments, and at least one parent had to be available for intervention sessions.

### Study Sample

**Fig. 2:**
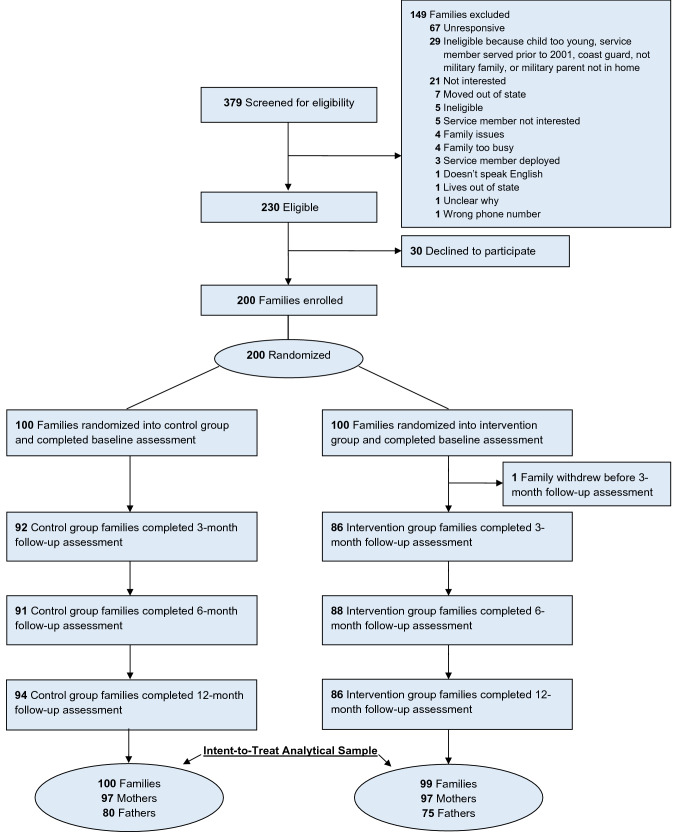
CONSORT diagram. Families were determined to have completed each respective assessment if at least one family member completed the assessment

Of 379 families screened for eligibility, 149 were excluded (see Fig. 2). Among the remaining 230 families, 30 declined to participate and 200 enrolled and were randomly assigned with equal allocation across the intervention group (FOCUE-EC) and control condition (OPE). One family subsequently withdrew consent resulting in 100 control (97 mothers and 80 fathers) and 99 intervention (97 mothers and 75 fathers) families with baseline data.

### Measures

#### Demographic Characteristics

Parents reported on all demographic characteristics listed in Table 1. Number of household adults and children, income, marital and employment status, and highest level of education were reported and reconciled where necessary to create family-level characteristics. When parents reported different levels of income, employment or education, preference was given to the higher level. Numbers of deployments and months deployed were summed across all military service members in a family and across both combat and non-combat deployments.

**Table 1:** Demographic characteristics of the FOCUS-EC intervention and control groups

Characteristics	FOCUS-EC intervention (n = 99)	Control (n = 100)	Statistics	P value
**Child characteristics**				
Gender				
Female	44 (44.4)	58 (58.0)	χ_1_^2^ = 3.66	.06
Male	55 (55.6)	42 (42.0)		
Age in months, mean (SD)	53.6 (12.7)	53.8 (11.9)	*t*_197_ = 0.09	.93
**Parent and family characteristics**				
Participating parents				
Mother and father	73 (73.7)	77 (77.0)	χ_2_^2^ = 0.67	.72
Mother only	24 (24.2)	20 (20.0)		
Father only	2 (2.0)	3 (3.0)		
Participating parent military status				
1 service member, 1 civilian parent	61 (61.6)	63 (63.0)	χ_2_^2^ = 0.36	.83
2 service members	12 (12.1)	14 (14.0)		
1 service member	26 (26.3)	23 (23.0)		
Participating parent role and military status			χ_4_^2^ = 2.84	.58
1 service member father, 1 civilian mother	59 (59.6)	63 (63.0)		
1 service member mother, 1 civilian father	2 (2.0)	0 (0.0)		
1 service member father, 1 service member mother	12 (12.1)	14 (14.0)		
1 service member father	2 (2.0)	3 (3.0)		
1 service member mother	24 (24.2)	20 (20.0)		
Number people in household				
1–3	28 (28.3)	24 (24.0)	χ_2_^2^ = 0.49	.78
4	32 (32.3)	35 (35.0)		
5 or more	39 (39.4)	41 (41.0)		
Number of children in household				
0	0 (0.0)	2 (2.0)	χ_5_^2^ = 6.71	.24
1	30 (30.3)	18 (18.0)		
2	39 (39.4)	46 (46.0)		
3	20 (20.2)	25 (25.0)		
4	9 (9.1)	7 (7.0)		
5 or more	1 (1.0)	2 (2.0)		
Number of children in household ages 0–4 years				
0	25 (25.3)	20 (20.0)	χ_4_^2^ = 2.20	.70
1	48 (48.5)	52 (52.0)		
2	21 (21.2)	25 (25.0)		
3	4 (4.0)	3 (3.0)		
4 or more	1 (1.0)	0 (0.0)		
Number of children in household ages 5–12 years				
0	39 (39.4)	33 (33.0)	χ_3_^2^ = 1.93	.59
1	31 (31.3)	39 (39.0)		
2	24 (24.2)	25 (25.0)		
3 or more	5 (5.1)	3 (3.0)		
Number of children in household ages 13–17 years				
0	92 (92.9)	88 (88.0)	χ_2_^2^ = 1.75	.42
1	5 (5.1)	10 (10.0)		
2	2 (2.0)	2 (2.0)		
Marital status				
Married or committed relationship	85 (85.9)	83 (83.8)	χ_1_^2^ = 0.16	.69
Other	14 (14.1)	16 (16.2)		
Maternal Race				
American Indian or Alaska Native	0 (0.0)	1 (1.1)	χ_6_^2^ = 7.17	.31
Asian	7 (7.5)	9 (9.7)		
Black or African American	7 (7.5)	8 (8.6)		
Native Hawaiian or Other Pacific Islander	3 (3.2)	0 (0.0)		
White	66 (71.0)	58 (62.4)		
Other	5 (5.4)	11 (11.8)		
More than one race	5 (5.4)	6 (6.5)		
Paternal Race				
Asian	2 (2.8)	4 (5.1)	χ_5_^2^ = 6.44	.27
Black or African American	4 (5.6)	9 (11.5)		
Native Hawaiian or Other Pacific Islander	2 (2.8)	0 (0.0)		
White	49 (68.1)	48 (61.5)		
Other	9 (12.5)	6 (7.7)		
More than one race	6 (8.3)	11 (14.1)		
Maternal Ethnicity				
Hispanic, Latino or Spanish origin	37 (38.5)	37 (39.0)	χ_1_^2^ = 0.00	.95
Not of Hispanic, Latino or Spanish origin	59 (61.5)	58 (61.1)		
Paternal Ethnicity				
Hispanic, Latino or Spanish origin	31 (42.5)	27 (34.2)	χ_2_^2^ = 1.13	.57
Not of Hispanic, Latino or Spanish origin	41 (56.2)	51 (64.6)		
Unknown	1 (1.4)	1 (1.3)		
Maternal age in years, mean (SD)	32.3 (5.4)	32.8 (4.6)	*t*_192_ = 0.76	.45
Paternal age in years, mean (SD)	33.5 (6.5)	33.4 (4.6)	*t*_*152*_ = *− 0.18*	.86
Employment				
Full-time	69 (69.7)	82 (82.0)	χ_1_^2^ = 4.11	.04
Part-time or less	30 (30.3)	18 (18.0)		
Income				
$39,999 or less	21 (21.4)	19 (19.2)	χ_2_^2^ = 0.87	.65
$40,000 to $59,999	27 (27.6)	23 (23.2)		
$60,000 or more	50 (51.0)	57 (57.6)		
Education				
Some college or less	52 (52.5)	46 (46.0)	χ_1_^2^ = 0.85	.36
Bachelor’s degree or more	47 (47.5)	54 (54.0)		
Military service classification				
Active duty	49 (49.5)	55 (55.0)	χ_2_^2^ = 0.62	.73
Veteran	35 (35.4)	32 (32.0)		
Guard or Reserve	15 (15.2)	13 (13.0)		
Maternal military service classification				
Active duty	8 (8.3)	6 (6.3)	χ_3_^2^ = 1.49	.68
Veteran	23 (23.7)	18 (18.8)		
Guard or Reserve	7 (7.2)	10 (10.4)		
Civilian	59 (60.8)	62 (64.6)		
Paternal military service classification				
Active duty	43 (58.1)	52 (65.8)	χ_3_^2^ = 2.72	.44
Veteran	20 (27.0)	21 (26.6)		
Guard or Reserve	10 (13.5)	6 (7.6)		
Civilian	1 (1.4)	0 (0.0)		
Total count of deployments				
0	12 (12.6)	9 (9.1)	χ_2_^2^ = 0.68	.71
1–2	30 (31.6)	31 (31.3)		
3 or more	53 (55.8)	59 (59.6)		
Total months of deployment, mean(SD)	20.5 (19.4)	20.2 (18.4)	*t*_191_ = *− *0.08	.93

Data presented as number (percentage) of participating families unless otherwise indicated

Differences in the reported n for some demographic characteristics were due to missing data

*SD* standard deviation

#### Parent Psychological Well-Being

Parent anxiety and depression symptoms were assessed using the Brief Symptom Inventory–18 (BSI-18; [38]). Parents rated the extent to which they were bothered by each symptom in the past week on a 0 (“Not at all”) to 4 (“Extremely”) Likert scale. Anxiety and depression scores were calculated by averaging the six items pertaining to each subscale (Cronbach’s α = 0.88, 0.89, respectively). Scores were calculated when no more than two items were missing per subscale. Missing items were imputed with the rounded mean score of the subscale’s non-missing items. The BSI-18 was administered at baseline, 3-, 6-, and 12-month assessment time points. Parent PTSD symptoms were assessed using the four-part Posttraumatic Diagnostic Scale (PDS; [39]). This study used the 17-item symptom severity section, which was completed by parents who indicated having lived through or witnessed a very stressful or traumatic event at some point in their lives. In this section, parents rated how frequently they were bothered by each symptom in the past month on a 0 (“Not at all or only one time”) to 3 (“5 or more times a week/almost always”) Likert scale. Re-experiencing and Arousal scores were calculated by summing the five items pertaining to each subscale, and Avoidance scores by summing the seven items pertaining to the subscale (Cronbach’s α = 0.94, 0.92, and 0.92, respectively). Total scores were calculated by summing all 17 items (Cronbach’s α = 0.96). Scores were calculated when no items were missing. The PDS, which has high internal consistency and test–retest reliability [39], was administered at baseline, 3- and 6-month assessment time points.

Parenting stress was assessed using the 36-item Parenting Stress Index–Short Form (PSI-SF; [40]). The PSI-SF consists of three subscales: Parental Distress, Parent–Child Dysfunctional Interaction (PC-DysFx), and Difficult Child. Most items of the PSI-SF were on a 1 (“Strongly Agree”) to 5 (“Strongly Disagree”) Likert scale; summed scores were calculated when no items were missing. The PSI-SF was administered at baseline, 3-, 6-, and 12-month assessment time points. The Parental Distress subscale examines the degree of distress parents are experiencing in their parental role and was considered as a measure of parent psychological health (Cronbach’s α = 0.89).

#### Parent–Child Relationships

Measurement of parent–child relationships consisted of both parent-reported and observational measures. Parents’ perceptions of their sensitive parenting behaviors were assessed using nine items from the Parental Behavior with Preschooler Q-Sort, which were adapted as self-report questionnaire items (PBP-Sensitivity; [41]); the self-report items have shown predictive validity for a variety of child and parent characteristics [22, 41]. On the PBP-Sensitivity, parents reported their level of agreement/disagreement with statements about their parent–child interactions in the past four weeks on a 1 (“Strongly agree”) to 5 (“Strongly disagree”) Likert scale. A total score was calculated by summing all nine items (Cronbach’s α = 0.86). Higher scores indicate greater parental sensitivity. The PBP-Sensitivity was administered at baseline, 3-, 6-, and 12-month assessment time points. Additionally, the PSI-SF PC-DysFx subscale (Cronbach’s α = 0.87) was used to measure parents’ expectations for their child and satisfaction with their parent–child interactions. Observational measures of parent–child relationships came from the Three Bags Task [42], in which both mother- and father-child dyads participated during the baseline and 12-month assessments (order of dyads were counterbalanced, and each dyad within a family received different but comparable materials). The last 5 min of each videotaped task were coded by undergraduate students who were blind to information regarding randomization status and assessment time point, under the guidance of a postdoctoral fellow and the project director. The coding system, adapted from the NICHD Study of Early Childhood Care [43], included three child scales (positive affect, negative affect, and engagement) and five parent scales (positive affect, negative affect, interactiveness, sensitivity, and intrusiveness), each of which received a single score for the entire interaction on a 1 (behavior not expressed) to 7 (behavior expressed frequently, intensely, or both) scale. Prior to independently coding videos, coders were required to reach 85% agreement with a master coder prior to beginning to code on their own. The threshold that we used was consistent with previous studies using this coding system [44]. Coder reliability was maintained by randomly selecting 20% of interactions of each coder to be double-coded; consistency among coders was monitored over time and discrepancies were discussed at weekly coding meetings.

The child composite score was created by taking the mean of all three child ratings after reverse scoring negative affect, resulting in the Observed Child Affect and Behavior score. The parent composite was created by taking the mean of the four of the five parent ratings (positive affect, negative affect, interactiveness, and sensitivity) after reverse scoring negative affect, resulting in the Observed Parent Affect and Behavior score. In the preferred single factor model, the intrusiveness scale did not load strongly and was thus excluded. Inter-rater reliability was assessed by calculating intra-class correlation (ICC) based on an absolute agreement definition for each composite score. For maternal parent–child interaction data, ICC was 0.71 and 0.65 for Observed Child Affect and Behavior and 0.60 and 0.69 for Observed Parent Affect and Behavior at baseline and 12-month assessment time points, respectively, which are considered moderate (0.50 to 0.75; [45]) or moderate-to-substantial (0.61 to 0.80; [46]). For paternal parent–child interaction data, ICC was 0.63 and 0.79 for Observed Child Affect and Behavior and 0.45 and 0.80 for Observed Parent Affect and Behavior at baseline and 12-month assessment time points, respectively. These ICCs are considered moderate or good [45], with the exception of paternal Observed Parent Affect at baseline. The Observed Parent Affect and Behavior composite score was considered as a measure of Parenting and Parent–Child Relationships.

#### Child Behavior

Measures of child behavior included the Observed Child Affect and Behavior composite score from the parent–child interaction task and the PSI-SF Difficult Child subscale, which examines parents’ perceptions of the child as easy or difficult to manage.

### Statistical Analysis

Descriptive statistics (means, standard deviations, frequencies, and percentages, as appropriate) were calculated for baseline child, parent, and family characteristics among the FOCUS-EC intervention and control (OPE) groups. To examine differences between these two treatment groups, Pearson chi-square tests were conducted for categorical variables and two independent sample t-tests were conducted for continuous variables.

In evaluating intervention effectiveness, an intention-to-treat analysis was used including all consented and randomized families and comparing across randomized treatment group assignments. Three sets of linear mixed-effects models were used to evaluate intervention efficacy based on reports/observations of: all parents combined, mothers only, and fathers only. Models for repeated measures were used to account for assessment time point (baseline and 3-, 6-, and 12-month follow-ups) as a repeated, within-subject factor. Treatment group (FOCUS-EC intervention or OPE) and child gender were included as between-subject factors along with an interaction between treatment group and assessment time point, used to model the treatment effect at each of the 3-, 6-, and 12-month time points. The *all parents* models also included parent gender as a fixed effect and random intercepts estimated at the family-level to account for nesting of participants within families. Child gender was included as a fixed effect because previous research indicates child gender differences in many of the outcome measures of interest [47, 48]. Consistent with the randomized design, other potential covariates were not included because they were not hypothesized a priori to impact intervention outcomes, and imbalances across treatment groups were not anticipated. A compound symmetry covariance structure was used to fit the block diagonal matrix for the random subject effects. Based on the fitted models, linear contrasts were constructed for each measure to estimate treatment effects relative to baseline at each of the 3-, 6-, and 12-month assessment time points and to test whether these effects differed significantly from zero. Observed parenting/child codes were collected only at baseline and 12 months. Linear mixed-effects models for these measures included a random intercept to account for repeated within-subject assessments along with an analogous set of fixed effects as described previously. As a measure of overall effect size, Cohen’s d was calculated for the difference between treatment groups in outcome changes from baseline to 12 months (6 months for PDS measures).

All statistical analyses were completed using the statistical software SAS, version 9.4 (SAS Institute Inc.). PROC MIXED was used to fit all linear mixed-effects models. In implementing PROC MIXED, the restricted maximum likelihood (REML) approach to estimation was invoked which accommodates data that are missing at random (MAR). This allowed for inclusion of all available data for each parent regardless of occasional missing data at the time point-level. The ESTIMATE statement with the CL option in PROC MIXED was also used to generate and test the statistical significance of the linear contrasts described previously. For all statistical testing performed, a significance level of 0.05 was used as the standard.

## Results

### Module Completion

Among families randomized to receive the FOCUS-EC intervention, 82 families (82%) completed at least four modules. Because the first four modules cover all of the core elements, families who completed at least four modules were considered as having completed FOCUS-EC for the purpose of this study. Seven (7%) families did not attend any sessions (typically due to deployments or moves), while ten families (10%) attended between one and three modules. The median number of days between sessions was 9 with mode of 7, corresponding to weekly visits. For the OPE condition, engagement with the asynchronous portal was modest, with 30 of the 100 (30%) control condition families accessing the portal 66 times.

### Demographic Characteristics

Family demographic characteristics are displayed in Table 1. The majority of families (75%) had two parents participating in the study; of these, 83% had one service member/veteran and one civilian parent and 17% were dual military parents. Of the 124 families with one service member/veteran and one civilian parent participant, 98% consisted of a service member/veteran father and civilian mother. Of the 49 families with a single service member participant, 90% consisted of a service member/veteran mother. Most families were married or in a committed relationship (85%). Three-quarters of families indicated two or more children currently living within the household. Eight percent of mothers and 9% of fathers identified as Black or African American, 67% of mothers and 65% of fathers identified as White, while 39% of mothers and 38% of fathers were of Hispanic, Latino, or Spanish origin. Eighty-nine percent of families reported at least one deployment with 58% reporting three or more. Parent and family characteristics in Table 1 did not differ significantly between conditions with the exception of employment status for which a lower percentage of families in the FOCUS-EC group reported full-time employment of at least one parent (70% vs. 82%, *p* = 0.04). Participating children ranged in age from 36 to 77 months at baseline (mean [M] = 53.7, standard deviation [SD] = 12.2). Fifty-six percent of children in the FOCUS-EC group and 42% of children in the OPE group were male (*p* = 0.06).

Analyses comparing parents stratified by parent role revealed no significant differences between conditions for mother-reported measures. Among father-reported measures, the only significant mean difference was BSI Depression (*p* = 0.03) which was higher among the OPE group. Means and standard deviations for parent-reported measures at baseline among the FOCUS-EC and control groups for mothers and fathers can be found in Table 2. Among the sample including all parents, there were no significant mean differences between groups at baseline.

**Table 2:** Mother- and father-reported mean scores and clinically meaningful symptoms at baseline

Measures at baseline	Mother-reported		Father-reported	
	FOCUS-EC intervention (N = 97)	Control (N = 96)	FOCUS-EC intervention (N = 74)	Control (N = 79)
**Parent psychological health**				
Anxiety, mean (SD)	0.38 (0.58)	0.39 (0.61)	0.31 (0.64)	0.52 (0.77)^Ɨ^
Clinically meaningful anxiety, n (%)	13 (13.4)	13 (13.5)	10 (13.5)	19 (24.1)^Ɨ^
Depression, mean (SD)	0.41 (0.65)	0.32 (0.48)	0.28 (0.55)	0.52 (0.80)^*^
Clinically meaningful depression, n (%)	11 (11.3)	5 (5.2)	14 (18.9)	25 (31.7)^Ɨ^
PTSD total, mean (SD)	8.05 (10.14)	7.62 (10.96)	8.13 (12.73)	11.73 (13.87)
Clinically meaningful PTSD^a^, n (%)	20 (26.7)	19 (26.0)	17 (25.4)	25 (35.7)
Re-experiencing^a^, mean (SD)	2.20 (3.09)	2.29 (3.29)	1.85 (3.68)	2.96 (4.01)^Ɨ^
Avoidance^a^, mean (SD)	2.88 (4.11)	2.70 (4.44)	2.82 (5.16)	4.41 (6.09)
Arousal^a^, mean (SD)	2.97 (3.87)	2.63 (3.96)	3.46 (4.75)	4.24 (4.49)
Parental distress, mean (SD)	25.39 (8.48)	26.14 (9.80)	22.97 (8.41)	24.13 (10.10)
**Parent–Child relationships**				
Parent–Child dysfunctional interaction, mean (SD)	19.80 (6.94)	19.17 (6.89)	18.74 (5.92)	18.95 (8.14)
Sensitive parenting, mean (SD)	35.28 (5.81)	35.67 (6.11)	36.04 (5.81)	36.04 (7.17)
**Child behavior**				
Difficult child, mean (SD)	24.96 (8.59)	24.02 (8.64)	23.00 (8.13)	22.73 (8.94)

Sample sizes varied slightly relative to those listed in the column headers due to item-level missing-ness (range: ≤ 5 fewer than column header value listed)

*SD* standard deviation

***p < 0.001; **p < 0.01; *p < 0.05; ^Ɨ^*p* < 0.10 for the comparison between FOCUS-EC and Control groups conducted for the baseline time point only

^a^Sample sizes differed substantially from those listed in the column headers. For the FOCUS-EC Intervention group: n = 75 for mothers, n = 67 for fathers. For the Control group: n = 73 for mothers, n = 72 for fathers

*p* < 0.05

Consistent with recruitment of a non-clinical, prevention sample, study parents reported relatively few psychological health symptoms (Table 2). Notably, the percentages of clinically significant scores did not differ between the FOCUS-EC and OPE groups on any scale for mothers or fathers (*p*s > 0.05).

### Intervention Effect on Parent Psychological Health

Compared to parents in the OPE group, FOCUS-EC parents reported greater reductions in PTSD symptoms from baseline to 6-months, including symptoms of re-experiencing and arousal with effect sizes [ES] = 0.44, 0.45, and 0.36, respectively (see Table 3). When analyzing mother and father subsamples separately, these significant results were only observed among mothers. Compared to mothers in the OPE group, FOCUS-EC mothers reported greater reductions in PTSD symptoms including symptoms of re-experiencing, avoidance, and arousal (ES = 1.06, 0.75, 0.74, and 0.67 respectively). There was no difference between groups on change in parent depression and anxiety symptoms or parental distress.

**Table 3:** Estimated intervention effects based on models including all parents, mothers only, and fathers only: intention-to-treat analysis

	All parents (N = 349)				Mothers only (N = 194)				Fathers only (N = 155)			
	BL to 3 M	BL to 6 M	BL to 12 M	*d*^a^	BL to 3 M	BL to 6 M	BL to 12 M	*d*^a^	BL to 3 M	BL to 6 M	BL to 12 M	*d*^a^
**Parent psychological health**												
Anxiety	* − *0.09	* − *0.05	* − *0.00	0.00	* − *0.05	0.03	0.06	0.14	* − *0.14	* − *0.16Ɨ	* − *0.08	0.14
Depression	* − *0.08	* − *0.05	* − *0.01	0.01	* − *0.01	0.02	0.09	0.20	* − *0.16	* − *0.14	* − *0.14	0.21
PTSD total^b^	0.52	2.78*	–	0.44	2.18Ɨ	4.40***	-	1.06	* − *1.81	0.75	–	0.10
Re-experiencing^b^	0.02	0.80*	–	0.45	0.82	1.30**	-	0.75	* − *0.98	0.15	–	0.14
Avoidance^b^	* − *0.09	0.82	–	0.28	* − *0.05	1.17*	-	0.74	* − *0.29	0.38	–	0.06
Arousal^b^	0.33	1.05*	–	0.36	1.01	1.41*	-	0.67	* − *0.62	0.52	–	0.10
Parental distress	* − *0.27	* − *0.09	1.43	0.20	0.14	-0.57	1.55	0.27	* − *0.64	0.71	1.39	0.13
**Parent–Child relationships**												
Parent–Child dysfunctional interaction	0.22	0.72	1.12	0.20	1.09	0.86	1.81*	0.36	* − *0.78	0.61	0.29	0.00
Sensitive parenting	* − *1.19Ɨ	* − *1.80**	* − *1.27Ɨ	0.21	* − *1.88*	* − *1.61*	* − *1.61*	0.34	* − *0.39	* − *2.09Ɨ	* − *0.88	0.10
Observed parent affect and behavior	–	–	* − *0.38***	0.39	-	-	* − *0.37**	0.43	-	-	* − *0.38*	0.34
**Child behavior**												
Difficult child	* − *0.01	0.91	1.43*	0.20	0.69	1.81Ɨ	2.09*	0.30	* − *0.82	* − *0.19	0.60	0.07
Observed child affect and behavior	–	–	− 0.33**	0.40	–	–	*− *0.37**	0.42	–	–	* − *0.29Ɨ	0.38

Intervention effect estimates are such that positive values indicate *greater decreases* from BL to follow-up among the intervention versus control group. Negative values indicate *greater increases* from BL to follow-up among the intervention versus control group

*BL* baseline, *3 M* 3-month follow-up, *6 M* 6-month follow-up, *12 M* 12-month follow-up, *d* Cohen’s d

***p < 0.001; **p < 0.01; *p < 0.05; ^Ɨ^*p* < 0.10

^a^Cohen’s d is calculated based on the change from BL to 6 M for the PDS measure as not administered at 12 months. For all other measures, calculations are based on the change from BL to 12 M

^b^The PDS was only asked among parents who indicated having lived through or witnessed a very stressful or traumatic event at some point in their lives, thus maximum sample size for PDS measures was 293 for All Parents models, 152 for Mothers Only models, and 141 for Fathers Only models

### Intervention Effect on Parent–Child Relationships

At 12-months, both mothers and fathers randomized to receive FOCUS-EC demonstrated significantly greater improvement in Observed Parent Affect and Behavior during the parent–child interactions, as compared to mothers and fathers in the OPE group (ES = 0.43 and 0.34, respectively). Compared to mothers in the OPE group, FOCUS-EC mothers reported significantly greater improvements in Sensitive Parenting from baseline to 3-, 6-, and 12-months (ES at 12-months = 0.34). At 12-months, FOCUS-EC mothers also reported significantly greater reductions in PC-DysFx (ES = 0.36).

### Intervention Effect on Child Behavior

Compared to parents in the OPE group, FOCUS-EC parents reported greater reductions in Difficult Child behavior from baseline to 12-months (ES = 0.20). When analyzing mother and father subsamples separately, these significant results were only observed among mothers (see Table 3). Observed Child Affect and Behavior improved to a greater extent from baseline to 12-months among FOCUS-EC families versus OPE families for the entire set of parent–child interaction tasks (ES = 0.40) and in particular for mother–child dyads (ES = 0.42), while this effect for father-child dyads was marginally significant (ES = 0.38).

## Discussion

The current randomized control trial indicates that the virtual delivery of FOCUS-EC resulted in sustained, positive effects on parent psychological health, parent–child relationships, and child behavior in MCF with 3- to 6-year-olds. Importantly and relevant to the current climate of the COVID-19 pandemic, these results support the efficacy of in-home virtual delivery of a trauma-informed family centered preventive intervention, a finding that can reduce barriers to care for military and non-military families alike.

Compared to parents randomized to the control group (i.e., OPE), parents in the FOCUS-EC group showed greater improvements in PTSD symptoms, but not depression or anxiety symptoms. This may be due to the specific targeting of parental PTSD symptoms, including trauma-informed psychoeducation and skills for managing trauma and loss reminders in the FOCUS-EC design. Notably, the intervention effect on PTSD symptoms was primarily within mothers. This may be accounted for by the more frequent attendance of mothers than fathers at FOCUS-EC sessions, even in two-parent families. Among the 91% (n = 477) of visits for which family member participation was recorded, only 194 sessions (41%) were attended by two parents; of the 283 sessions attended by only one parent, the vast majority (88%, n = 249) were attended by a mother only. Thus, even in families in which two parents participated in the research study, mothers may have been more likely to benefit from participation in the intervention.

A study of another parenting program for MCF found similar patterns of effects on PTSD symptoms for mothers but not fathers; the authors interpreted that finding in the context of fathers in their sample having significantly higher levels of PTSD symptoms than mothers [49]. This was not the case in the current sample, in which PTSD symptom levels were comparable for mothers and fathers. Taken together, the findings from these two studies underscore the broad range of risk and resilience patterns that may be found in MCF. Additionally, more than half (58%) of the mothers in this sample who reported experiencing a traumatic event were not service members or veterans themselves, suggesting that PTSD symptoms stemmed from non-military traumatic events. Thus, it is critical that providers serving military families understand the multitude of potentially traumatic events that parents may face even outside of the military experience.

FOCUS-EC families also showed greater improvement than OPE families in parent-report and observed measures of parents’ and children’s behaviors and emotions. Similar to the intervention effect on PTSD symptoms, mothers were the primary drivers of the parenting and child effects. When examining the subsample of mothers, there were significant intervention effects not only for observed maternal affect and behavior during the parent–child interaction task, but also for observed child affect and behavior, and maternal reports of their perceived sensitive parenting, parent–child interaction quality, and child’s behavior problems. When examining a subsample of just fathers, however, the only significant intervention effect was for observed father affect and behavior. Because fathers participated in the FOCUS-EC sessions less frequently than mothers, it is possible that they learned behavior that was modeled by the mother but did not perceive a change in their own parenting because they did not receive the developmental guidance to explain why the parenting behavior was important. It is also possible that improvement in child behavior over the course of the intervention resulted in increased overall positivity in the father-child dyad; in other words, if child behaviors were more positive, it would be easier for fathers to respond in a positive, child-centered manner.

It is notable that intervention effects for both mother and child affect and behavior were found across both parent-reported and observational measures. Although the broad body of research on family and child development routinely incorporates observational measures of parent–child interaction into their study designs, the vast majority of military family research relies on parent- and child-report (for exceptions see [28, 50]). That parents receiving FOCUS-EC (as compared to parents in OPE) demonstrated greater improvements in *observed* behaviors during parent–child interactions provides stronger support for FOCUS-EC intervention efficacy than parent-report measures alone, because they obviate the possibility of shared method variance. These findings also hold for child outcomes, as children in the FOCUS-EC group showed greater positive engagement during parent–child interaction tasks and were perceived to be less difficult by their mothers than children in OPE group. Notably, reliance on father self-report measures in this study would have obscured changes in behaviors that were evident when observed during actual parent–child interaction.

Unlike prior intervention studies with military families (ADAPT, SFSF), the FOCUS-EC intervention was designed not only for families that recently experienced a deployment, but more broadly for MCF experiencing the full range of military life transitions. For example, families may experience ongoing military-related stressors for years after a deployment, such as repeated family separations due to military trainings, transitioning from active duty to veteran status, and navigating services in civilian communities. FOCUS-EC’s demonstrated efficacy in a sample that was heterogenous in terms of duty status and length and recency of deployments suggests that the program is beneficial for a wide range of military families.

There are important implications for military- and veteran-serving systems, in that positive effects were found with virtual delivery of a preventive home visiting intervention. MCF, particularly Reserve Component and Veteran families, face multiple barriers to accessing military-informed care, including geographic dispersion, transitions in care systems (e.g., DOD to VHA), lack of family services within VHA, and financial challenge [51]. Some interventions have addressed barriers through in-person home visiting models [29], but home visiting can be expensive, with average annual cost per family estimated at $8497 [52], and is not always feasible across a widely dispersed population. In-home virtual delivery may also prove useful for engaging families separated by distance, such as for officer school or operational missions. That session adherence rates in this study were high (82% attended five or more sessions) suggests that home visiting via a telehealth platform is feasible and acceptable to this population of families with young children. The impact of FOCUS-EC delivered virtually is especially notable given that this study utilized an active control group that also accessed an online platform containing high-quality parenting educational resources typically used to support MCF. Notably, parents in the OPE condition had somewhat limited engagement with the self-directed educational resource modules. Fortunately, with the advancement of more interactive technologies, virtual interventions have the potential to yield positive effects while circumventing obstacles to care and still benefiting from live engagement.

### Limitations

Despite the promising results, our sample size may have limited the ability to identify additional intervention effects. While the heterogeneity in the sample can be viewed as a strength, the inclusion of active duty, reserve component and veteran families may have limited our ability to precisely characterize the sample and intervention effects and has implications for generalizability. Future studies should consider assessing impact among specific populations of MCF (veteran, active duty, ethnic/racial minorities, etc.), which could drive future development of culturally responsive interventions, such as those for ethnically and racially diverse MCF as have been undertaken for FOCUS in other settings [53]. Awareness of the limited sample size available impacted our selection of covariates to include in statistical models. A small number of covariates were selected a priori, as is advisable for randomized trials [54], with the acknowledgement that additional factors that could have influenced intervention effectiveness were omitted and remain a topic to be addressed by future studies. As noted above, session attendance was relatively low in fathers, which may have limited our ability to assess the direct impact of intervention participation on father outcomes. Inter-rater reliability was poor for one of the composite scores used in outcome analyses (paternal Observed Parent Affect at baseline). After conducting post-hoc exploratory analyses to understand the relatively low ICC (0.45), we determined that the subset of data that was double-coded for reliability had a more restricted range than the rest of the observed parent data (including full sample of father data at baseline, mother baseline, and father follow-up). Restriction of range is known to result in lower ICCs [55]. Given that the full sample of father baseline data had a wider range, and the adequate-to-excellent ICCs for all other codes, we chose to move forward with planned analyses for all observed codes. Further, we anticipate that this decision likely had a conservative impact on inferences due to reduced power attributable to the potentially high measurement error. Notably, the parents in this study had relatively low levels of depression and anxiety compared to those reported previously for active duty families seeking family-prevention services [32]. Help-seeking families may benefit in different ways or to a different extent relative to the actively-recruited families in this study which may have limited our ability to identify impact on parental psychological health symptoms relevant to a more distressed population of MCF.

### Clinical Implications

The results underscore the importance of trauma-informed family preventive intervention models for MCF with young children in the context of military life transitions, including deployment related experiences. Just as stress and adversity can reverberate across the family system, so too can resilience. By engaging the entire family, FOCUS-EC addresses parental psychological well-being and parent–child relationships through a family systems model. In addition to providing developmental and parenting guidance, the intervention provides the opportunity to learn and practice core resilience skills, such as family communication and managing trauma reminders. Further, family narrative and emotion regulation activities promote the parents’ ability to reflect on the thoughts, emotions, and experiences of their child from a developmental lens, with potential benefits for parent–child relationships.

The results presented also have broader implications for healthcare policy and implementation practices. These findings may have relevance for any population that faces barriers to receiving in-person behavioral health care, including individuals living in rural areas, those with limited transportation options, non-traditional work schedules, physical disabilities, fragile medical conditions, or living in communities in which mental health care is stigmatized. At time of this writing, the world is facing a global pandemic (i.e., COVID-19) that is causing many behavioral health providers to quickly adopt new strategies [56] to serve children and families. This study adds to a small but growing literature supporting in home virtual behavioral health delivery for parents and children (e.g., internet-based parent child interaction therapy; [57]) and provides hope that existing models can be successfully adapted for remote delivery.

## Summary

Stressors associated with military life may negatively affect child development, parent psychological well-being, and parent–child and family relationships. This randomized controlled trial evaluated the efficacy of Families OverComing Under Stress-Early Childhood (FOCUS-EC), a trauma-informed and family-centered preventive intervention designed to promote family resilience, effective parenting, positive parent–child relationships, and psychological well-being in the context of stress and adversity. FOCUS-EC was delivered via virtual home visiting to enhance ecological validity as well as access to care for military-connected families with young children who might have difficulty obtaining traditional clinic-based services.

Military connected families with 3- to 6-year-old children (n = 199 families; 194 mothers; 155 fathers; 199 children) were randomized to the FOCUS-EC intervention condition (n = 99) or online parent education control condition (n = 100). Parent psychological health symptoms, child behavior, parenting and parent–child relationships were examined by parent-report (at baseline, 3, 6, and 12 months) and observed interaction tasks (baseline and 12 months). Longitudinal regression models showed that FOCUS-EC families demonstrated significantly greater improvements at 12 months in parent-reported difficult child behavior, mother-reported parenting practices, observed child affect and behavior, and observed parent affect and behavior relative to families receiving an online parent education program. FOCUS-EC parents also demonstrated greater reductions in posttraumatic stress symptoms at 6 months.

Thus, FOCUS-EC had a sustained positive effect on parent PTSD symptoms, parent–child relationships, and child behavior in military connected families with 3- to 6-year-olds. Telehealth delivery of FOCUS-EC in this study indicates the potential for in-home virtual delivery of preventive interventions for families with young children facing adversity.
